# Defective Artemis causes mild telomere dysfunction

**DOI:** 10.1186/2041-9414-1-3

**Published:** 2010-05-26

**Authors:** Hemad Yasaei, Predrag Slijepcevic

**Affiliations:** 1 Brunel Institute of Cancer Genetics and Pharmacogenomics, Division of Biosciences, School of Health Sciences and Social Care, Brunel University, Uxbridge, Middlesex, UB8 3PH, UK

## Abstract

**Background:**

Repair of DNA double strand breaks by non-homologous end joining (NHEJ) requires several proteins including Ku, DNA-PKcs, Artemis, XRCC4, Ligase IV and XLF. Two of these proteins, namely Ku and DNA-PKcs, are also involved in maintenance of telomeres, chromosome end-structures. In contrast, cells defective in Ligase IV and XRCC4 do not show changes in telomere length or function suggesting that these proteins are not involved in telomere maintenance. Since a mouse study indicated that defective Artemis may cause telomere dysfunction we investigated the effects of defective Artemis on telomere maintenance in human cells.

**Results:**

We observed significantly elevated frequencies of telomeric fusions in two primary fibroblast cell lines established from Artemis defective patients relative to the control cell line. The frequencies of telomeric fusions increased after exposure of Artemis defective cells to ionizing radiation. Furthermore, we observed increased incidence of DNA damage at telomeres in Artemis defective cells that underwent more than 32 population doublings using the TIF (Telomere dysfunction Induced Foci) assay. We have also inhibited the expression levels of DNA-PKcs in Artemis defective cell lines by either using synthetic inhibitor (IC86621) or RNAi and observed their greater sensitivity to telomere dysfunction relative to control cells.

**Conclusion:**

These results suggest that defective Artemis causes a mild telomere dysfunction phenotype in human cell lines.

## Background

There is increasing evidence that the maintenance of telomeres, physical ends of chromosomes, and DNA damage response mechanisms are interlinked. The first observation of a telomere dysfunction phenotype in a DNA damage response defective environment was reported in the case of Ataxia telangiectasia (AT) cells. The telomere dysfunction phenotype in cells from AT patients or ATM (AT mutated) defective mice ranges from accelerated telomere shortening to end-to-end chromosome fusions and extra-chromosomal telomeric fragments [1,2]. Following the observation of telomere dysfunction associated with the ATM defect, a number of DNA damage response factors have been shown to affect telomere maintenance. Most notably, proteins involved in the repair of DNA double strand breaks (DSBs) either by Non-Homologous End Joining (NHEJ) or homologous recombination (HR) including Ku, DNA-PKcs, RAD54, RAD51D and BRCA1 if dysfunctional, will cause a severe telomere dysfunction phenotype [3-6]. So far, at least 17 DNA damage response proteins have been shown to affect telomere maintenance [7]. It is not yet clear as to why the interplay between telomere maintenance and DNA damage response is required. However, it is certain that both pathways are essential for chromosome integrity maintenance and perhaps their interaction is important for the stable chromosome segregation.

One of the key pathways required for the stable segregation of chromosomes is NHEJ. The key players in this pathway are Ku 70/86 and DNA-PKcs, both shown to be involved in telomere maintenance [3]. Other proteins involved in NHEJ include: Artemis, Ligase IV, XRCC4 and XLF [8]. Previous studies have shown that Ligase IV and XRCC4 do not have effect on telomere length or function [9]. However, it is not clear yet whether the remaining two NHEJ proteins, namely Artemis and XLF, affect telomere maintenance.

Artemis has exonuclease and endonuclease activities in the presence of DNA-PKcs and ATP [10]. It is required for V(D)J recombination and people with mutations in the gene coding for Artemis show immunodeficiency and radiosensitivity [11]. Thus, the human disease due to defective Artemis is named RS-SCID (radio-sensitive severe combined immunodeficiency disease).

A study of cells from Artemis defective mice [12] revealed slightly elevated frequencies of end-to-end chromosome fusions, a cytological sign of telomere dysfunction. Furthermore, analysis of a primary fibroblast cell line from an RS-SCID patient showed accelerated shortening of telomeres relative to the normal control cell line [13]. These studies point to the possibility that Artemis, similarly to the other two NHEJ proteins, Ku and DNA-PKcs, may have a role in telomere maintenance. This possibility is further supported by observations that a close homologue of Artemis, a protein named Apollo, is directly involved in telomere maintenance, most likely true interactions with the telomeric protein TRF2 [14,15].

In this study we analyzed spontaneous and radiation induced chromosomal abnormalities and monitored repair kinetics of ionizing radiation (IR) induced DSBs occurring within telomeric sequences in Artemis defective human cells. Furthermore, we either inhibited or knocked-down DNA-PKcs and monitored the effect of this procedure on telomeres. Our results suggest that defective Artemis causes a mild telomere dysfunction phenotype.

## Results

### Spontaneous telomeric fusions in Artemis defective cells

**Figure 1:**
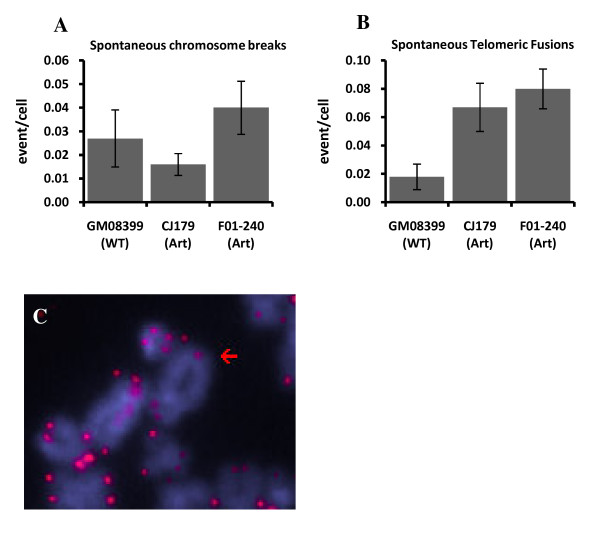
**Spontaneous chromosome abnormalities in Artemis defective cells**. A. Chromosome breaks. B. Telomeric fusions. C. Example of a telomeric fusion in a CJ179 cell: sister chromatid union. Error bars represent standard deviation (SD). Frequencies of abnormalities per cell were calculated from three separate experiments. The total number of metaphase cells analysed were: GM08399 - 111, CJ179 - 193 and F01-240 - 174.

We started by analyzing spontaneous chromosome abnormalities in two Artemis defective cell lines and a control cell line using FISH with the telomeric PNA (peptide nucleic acid) probe. The total number of end-to-end chromosome fusions (telomeric fusions) and chromosome breaks were recorded. No significant differences between frequencies of spontaneous chromosome breaks were observed between Artemis defective and control cells (Fig. 1A). However, we observed low but significantly higher frequencies of telomeric fusions in the two Artemis defective cell lines compared to the normal control cell line (p < 0.001) (Fig. 1B). The majority of these fusions were sister chromatid unions (~62%) (Fig. 1C). Given the significantly elevated level of telomeric fusions in Artemis defective cells relative to control cells, our results indicate that the Artemis defect may cause a mild telomere dysfunction phenotype in human cells.

### Radiation induced telomere dysfunction in Artemis defective cells

**Figure 2:**
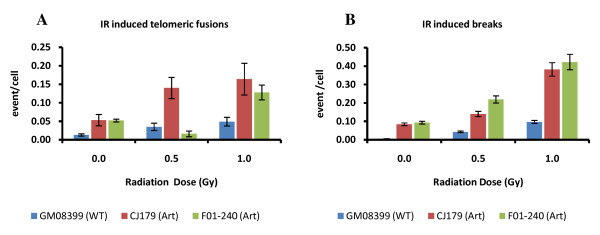
**IR induced telomeric fusions in Artemis defective cells**. A. Telomeric fusions. B. Chromosome breaks. Two doses of IR were used: 0.5 Gy and 1.0 Gy in three independent experiments. Over 100 metaphase cells were analysed per dose. The error bars represent SD.

We next examined whether telomere dysfunction is elevated following exposure of Artemis defective cells to ionizing radiation (IR). Interestingly, frequencies of telomeric fusions were significantly higher in Artemis defective cell lines than in the control cell line (p < 0.001) after irradiating cells with 1.0 Gy of gamma rays (Fig. 2A). The majority of telomeric fusions were of chromatid type (56%) but we also observed some chromosome type telomeric fusions i.e. dicentric chromosomes (30%). Only the CJ179 cell line showed statistically significant difference in telomeric fusions relative to the control line at both doses used (0.5 and 1.0 Gy) (Fig. 2A). Relative insensitivity of the F01-240 cell line to the formation of telomeric fusions at the dose of 0.5 Gy can be attributed to a type of mutation present in this cell line that may lead to quantitatively different effects at different doses. Therefore, our results suggest that Artemis defective cell lines show slightly but significantly elevated IR induced telomeric fusions in comparison to the control cell line. This further indicates the presence of a mild telomere dysfunction phenotype as a result of Artemis deficiency in human cells.

Our results have also confirmed that the two Artemis defective cell lines exhibit significantly higher frequencies of IR induced chromosome breaks compared to the normal control cell line (p < 0.001) (Fig. 2B). This is similar to published result [16].

### Analysis of Artemis defective human cell lines using immuno FISH

To confirm the presence of a mild telomere dysfunction phenotype in Artemis defective cell lines we used the modified version of the TIF (Telomere dysfunction Induced Foci) assay. This assay usually relies on simultaneous detection of telomeres (antibodies against TRF1 or TRF2) and DNA damage (antibody against a DNA damage marker) [17]. However, instead of antibodies against telomeric proteins we used telomeric PNA in combination with an antibody against a DNA damage marker, γ-H2AX (immuno-FISH). Given that telomeres shorten in proliferating primary human fibroblasts [18] and that this shortening can affect telomere function [19] we decided to analyze cells in relatively early passages ("younger" cells) as well as cells in late passages ("older" cells). Cells with PD (population doubling) value below 16 represented "younger" cells and cells with PD value of 32-33 represented "older" cells.

**Figure 3:**
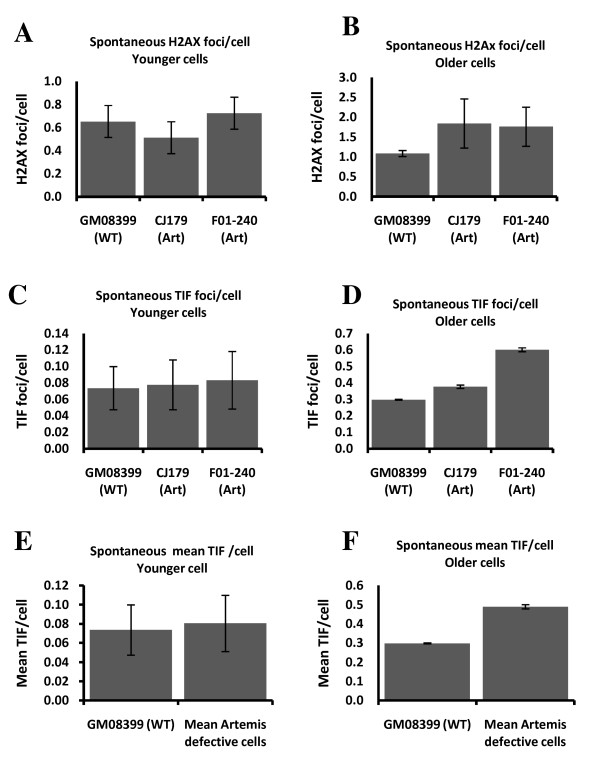
**Frequencies of spontaneous γ-H2AX positive foci and TIFs in "younger" and "older" cells**. A and B. Average γ-H2AX positive foci in each cell line. C and D. Average TIFs per in each cell line. E and F. Mean frequencies of TIFs from two Artemis defective cell lines pooled together. All results are based on four independent experiments with an average of 200 cell nuclei scored per experiments per cell line.

Artemis defective and normal "younger" cells had similar frequencies of spontaneous γ-H2AX positive foci (Fig. 3A) and this is in line with published results [20]. However, γ-H2AX positive spontaneous foci increased in Artemis defective and normal "older" cells relative to "younger" cells (Fig. 3B). Interestingly, the difference in spontaneous frequencies of γ-H2AX positive foci between Artemis defective and normal "older" cells was statistically significant (p < 0.001). This could mean that "older" Artemis defective cells are less efficient in repairing endogenous DNA DSBs than the normal control cells of the same age.

**Figure 4:**
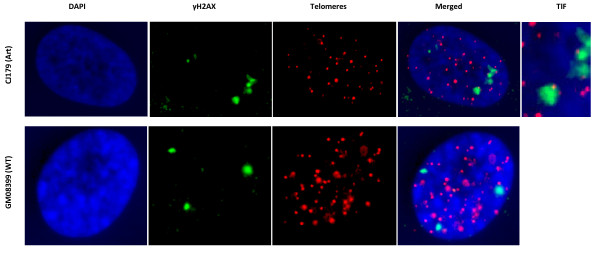
**Representative examples of cell nuclei with or without TIFs**. Upper panel: a cell nucleus showing a TIF. Lower panel: a cell nucleus with separate γ-H2AX and telomere signals (no TIF).

The immuno-FISH analysis showed no difference in spontaneous TIF frequencies between Artemis defective and normal "younger" cells (Fig. 3C and 3E). Therefore, the observed mild spontaneous telomere dysfunction in Artemis defective cells, which manifests as elevated level of telomeric fusions (Fig. 1B) cannot be linked to the results of immuno-FISH analysis. However, it is important to note that the average PD of cells used in experiments described in Fig. 1 was PD 24 (ranging from 19 to 32). Therefore, it is possible that the observed mild spontaneous telomere dysfunction occurs only in "older" cells. In line with this possibility "older" Artemis defective cells had a significantly higher frequency of spontaneous TIFs than normal "older" cells (Fig. 3D and 3F). Representative examples of TIFs are shown in Fig 4. Based on these results it seems reasonable to argue that telomere dysfunction in Artemis defective cells increases with PD number. When Artemis defective cells are relatively "young" (low PDs) they show functional telomeres. However, when Artemis defective cells become "older" (PD 32+) but not senescent yet, they show a small but significant increase in spontaneous TIF frequency relative to normal cells of similar age (Fig. 3D and 3F).

### Repair kinetics of IR induced DNA damage at telomeres

**Figure 5:**
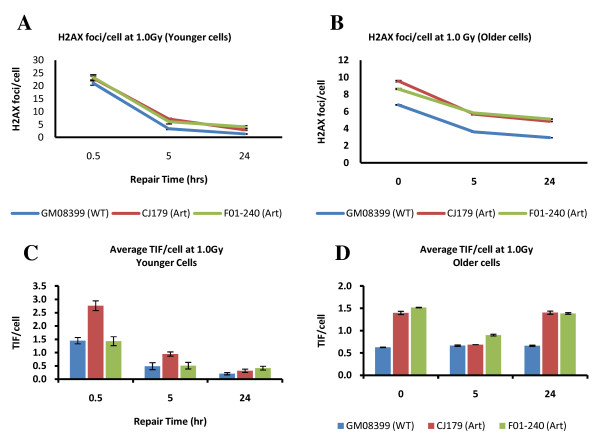
**Repair kinetics of IR induced DNA damage**. A and B. γ-H2AX foci per cell. C and D. TIFs per cell. Results are based on four independent experiments with on average 200 cell nuclei analysed per experiment per point. Error bars represent SEM. The dose of IR used was 1.0 Gy.

We next wanted to establish whether Artemis defective cells show changes in repair kinetics of DNA damage at telomeres relative to normal cells using the TIF assay. The TIF assay allows identification of all γ-H2AX positive foci in the nucleus, as well as γ-H2AX foci with overlap with telomeric DNA. Previous studies established that Artemis defective cells have impaired repair kinetics of DSBs relative to control cells when analyzed using γ-H2AX as a marker of DSBs [20-22]. Frequencies of γ-H2AX foci and TIFs after 1.0 Gy of gamma rays at different times after exposure are shown in Fig. 5. Artemis defective and normal "younger" cells showed expected kinetics of DSB repair. For example, "younger" Artemis defective cells had a higher proportion of unrepaired DSBs 24 h after IR relative to normal "younger" cells (Fig. 5A) in line with published studies [20-22]. Interestingly, frequencies of IR induced TIFs were similar in Artemis defective and normal "younger" cells 24 h after IR (Fig. 5C) suggesting that DNA damage at telomeres is repaired normally in Artemis defective "younger" cells.

However, "older" Artemis defective cells behaved differently as shown in Fig. 5B and 5D. Most importantly, normal and Artemis defective "older" cells showed higher proportion of unrepaired TIFs 24 h after IR relative to "younger" cells suggesting the reduced capacity to repair damage at telomeric DNA in these cells (Fig. 5D). It is important to note the differences in frequencies of γ-H2AX foci at the first point after IR between "older" and "younger" cells (Fig. 5). We processed "younger" cells for immuno-cytochemical analysis 0.5 h after IR, whereas "older" cells were processed immediately after IR. It is known that the frequencies of γ-H2AX foci peak approximately 30 min after IR [20]. This explains the lower frequencies of γ-H2AX foci immediately after IR in "older" cells relative to frequencies of γ-H2AX foci observed in "younger" cells 0.5 h after IR. Consequently the frequencies of TIFs are lower in "older" cells than in "younger" cells at the first point of measurement after IR. It is reasonable to assume that the level of TIFs in "older" cells should be at least the same as in "younger" cells if older cells are processed for analysis 0.5 h after IR. Therefore, the impression that no repair of DNA damage at telomeres takes place in older cells, judging by the same levels of TIFs at the first point of measurement after IR and 24 h after IR is wrong (Fig. 5D). It is likely that some repair within telomeric sequences in older cells takes place but it is less effective than in "younger" cells judging by the level of unrepaired TIFs 24 h after IR (Fig. 5C and 5D). It is clear that Artemis defective older cells are less able to repair TIFs than their normal counterparts. For example, normal "older" cells had approximately 0.6 TIFs/cell 24 h after IR, whereas the two Artemis defective cell lines had 1.4 and 1.5 TIFs/cell respectively 24 h after IR (Fig. 4A). When the level of spontaneous TIFs (0.3 in normal and 0.5 in Artemis defective "older" cells) is deducted from the above values the difference in the frequency of IR induced TIFs is approximately 3-4 times higher in Artemis defective "older" cells than in their normal counterparts 24 h after IR. It is interesting that Artemis defective cells have shown a dip in TIF frequencies 5 h after IR.

These results argue that (i) "older" cells cannot repair DNA damage at telomeres as efficiently as "younger" cells and (ii) the effect of inefficient repair of DNA damage at telomeres in older cells is stronger in Artemis defective cells than in their normal counterparts.

### DSB repair kinetics after DNA-PKcs inhibition

**Figure 6:**
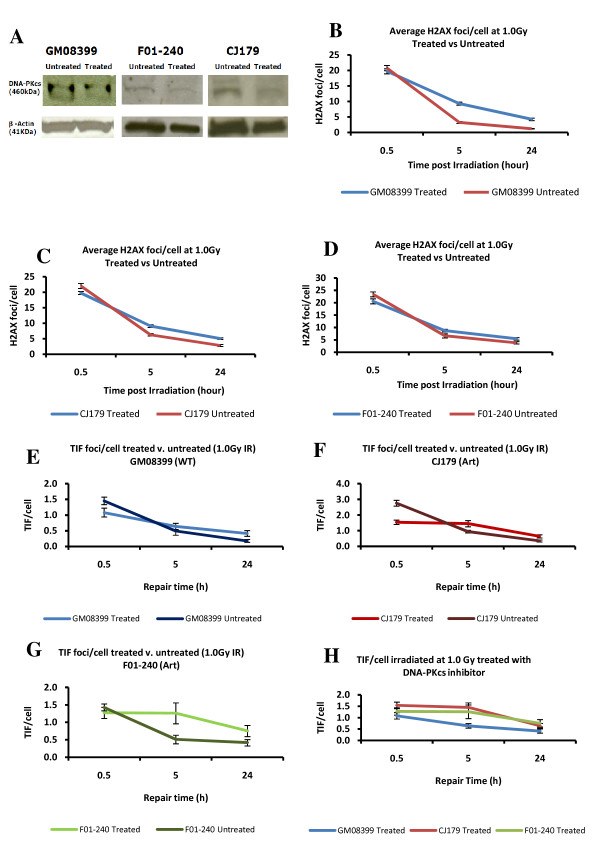
**Kinetics of DNA damage repair after DNA-PKcs inhibition**. A. Results of Western blotting analysis. A reduction of 45% in DNA-PKcs protein level was observed 24 hrs after treatment of cells with 20 μM/μl of IC86621 based on densitometry analysis (software used: ImageQuant V5.1). B-D. Frequencies of γ-H2AX positive foci per cell after 1.0 Gy of gamma rays before and after DNA-PKcsi treatment. E-H. Frequencies of TIF per cell after 1.0 Gy of gamma rays before and after DNA-PKcsi treatment. The results are based on three independent experiments with at least 100 cell nuclei analysed per experiment per point. Error bars represent SEM in B-D and SD in E-H. CJ179 and F01-240 are Artemis defective cell lines whereas GM08399 is a wild type cell line.

We next analysed DSB repair kinetics by the TIF assay in Artemis defective "younger" cells subjected to DNA-PKcs inhibition (DNA-PKcsi). DNA-PKcs is one of the proteins present at telomeres and its loss causes telomere dysfunction [23]. Furthermore, DNA-PKcs phosphorylates Artemis during the process of NHEJ [24]. To inhibit DNA-PKcs we used a synthetic DNA-PKcs inhibitor, IC86621. Previous studies have shown that IC86621 generates telomeric fusions in human and mouse cells via inhibiting DNA-PKcs [25,26]. Artemis defective and control cell lines were subjected to DNA-PKcsi (20 μM/μl of IC86621) for the period of 24 h and levels of DNA-PKcs were quantified by western blot (Fig. 6A). This treatment resulted in 45% reduction in DNA-PKcs levels in all three cell lines (Fig. 6A). We have also shown that DNA-PKcsi by 20 μM/μl of IC86621 for 24 h causes telomeric fusions in mouse cell lines (Yasaei and Slijepcevic, manuscript in preparation). After 24 h treatment with IC86621 we exposed cells to 1.0 Gy of gamma rays and monitored DSB repair kinetics. All cell lines used for this experiment were, on average, at PD 16 (range 11-17) (younger cells) at the time of irradiation. As expected DNA-PKcsi resulted in increased frequencies of γ-H2AX positive foci 24 h after IR in all cell lines (Fig. 6B). Analysis of TIFs showed that Artemis defective cells repair DSBs at telomeres in a slower fashion than control cells (Fig. 6C). The two Artemis defective cell lines subjected to DNA-PKcsi repaired only 1 - 6% of DSBs within telomeres compared to 41% repaired DSBs within telomeres in the normal cell line 5 h post IR (Fig. 6C). Twenty four h post IR the two Artemis cell lines had on average ~50% of unrepaired TIFs, whereas normal cells showed 38% of unrepaired TIFs (Fig. 6C). The difference in TIFs frequencies 24 hours post IR was statistically significant (i) between the two Artemis defective DNA-PKcsi treated and untreated cell lines and (ii) control treated and untreated cells (P < 0.001).

These results demonstrate two points. First, DNA-PKcsi caused elevated frequencies of unrepaired TIFs 24 hours post IR in both normal and Artemis defective cell lines relative to control cells which is in line with expectations from published studies. Second, DNA-PKcsi treated Artemis defective cell lines showed slower repair kinetics than normal cells, suggesting a greater sensitivity of Artemis defective cells to induced telomere dysfunction.

### Knock-down of DNA-PKcs expression by RNAi

**Figure 7:**
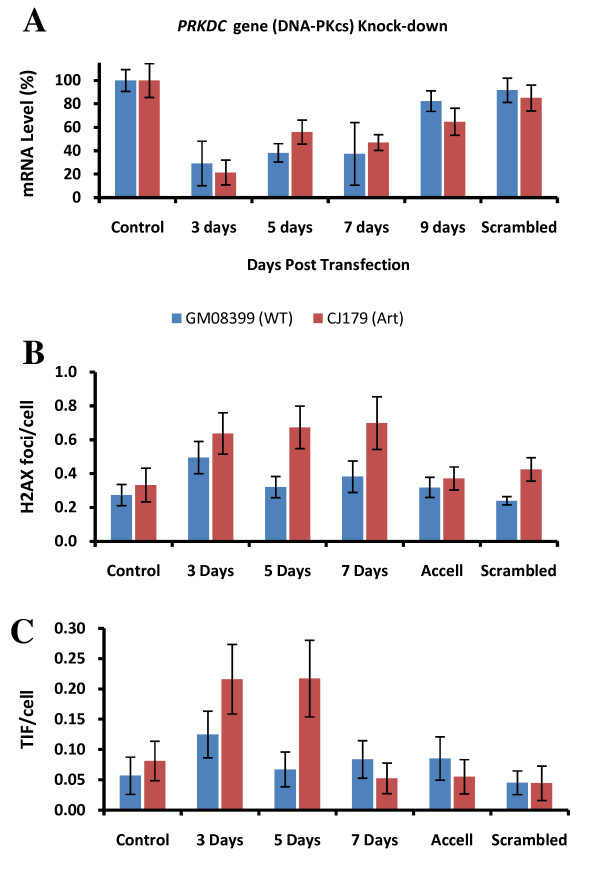
**PRKDC knock-down by RNAi**. A. Analysis of DNA-PKcs expression by real time PCR after transfection with siRNA oligonucleotides specific for *PRKDC*. The results were normalized against endogenous *GAPDH *mRNA expression and are based on two independent experiments. Non-targeting (scrambled) siRNA sequence was used as negative control. Error bars indicate SD. B. Frequenceis of γ-H2AX foci before and after transfection. The data are based on two independent experiments with an average of 100 nuclei scored per point. The error bars represent SEM. C. Frequencies of TIFs before and after transfection. The data are based on two independent experiments. Error bars represent SEM. The difference in TIF between the two lines is significant at 5 days post transfection (p < 0.012).

To confirm the greater sensitivity of Artemis defective cells to telomere dysfunction via reduction in DNA-PKcs expression levels we subjected an Artemis defective cell line, CJ179, and the control cell line to transfection with short interfering (si) RNA oligonuclotides specific for the gene encoding DNA-PKcs and monitored the level of DNA-PKcs expression by real time PCR for 9 days after transfection. The reduction in DNA-PKcs expression of approximately 70% - 80% was observed 3 days post-transfection (Fig. 7A). The reduction remained strong 5 and 7 days post-transfection followed by the recovery in DNA-PKcs expression 9 days post-transfection (Fig. 7A). The frequency of γ-H2AX foci increased in normal cells only 3 days after transfection whereas their level was normal 5 and 7 days after transfection (Fig. 7B). Interestingly, the effect was stronger in Artemis defective cells leading to significantly higher frequencies of γ-H2AX positive foci up to 7 days post-transfection. Similarly, frequencies of TIFs were higher in Artemis defective cells 3 and 5 days after transfection than in control cells (Fig. 7C). However, TIF frequencies returned to normal levels 7 days post-transfection. Therefore, these results confirm a greater sensitivity of Artemis defective cells to induced telomere dysfunction via DNA-PKcs knock-down relative to control cells.

## Discussion

The first evidence of chromosomal abnormalities in Artemis defective cells that result from telomere dysfunction was presented previously in mice [12]. The analysis of mouse ES cells that lack functional Artemis revealed higher frequencies of telomeric fusions in comparison to control cells. In contrast, two published studies that used Artemis defective human cell lines [16,27] reported no spontaneous or IR induced chromosomal abnormalities resulting from telomere dysfunction. However, these studies relied on the classical cytological analysis and were not designed to specifically probe for telomere dysfunction. The aim of our study was to employ telomere specific FISH with the purpose of identifying subtle chromosome abnormalities resulting from telomere dysfunction in Artemis defective human cell lines that cannot be detected by classical cytological methods. In line with previously published data [12] our results revealed a slight but significant increase in both spontaneous and IR induced telomeric fusions in Artemis defective cell lines relative to the control cell line (Figs 1B and 2A).

Mouse cells defective in two NHEJ proteins, namely Ku and DNA-PKcs, show high frequencies of telomeric fusions [3]. In many instances multiple telomeric fusions were present in individual Ku or DNA-PKcs defective cells [3,23]. In contrast, frequencies of telomeric fusions in Artemis defective mouse cells were much lower than in Ku or DNA-PKcs defective cells. For example, Rooney et al. (2003) found 8 spontaneous telomeric fusions in 130 analyzed mouse ES cells (0.06/cell). This is remarkably similar to our results. We found, on average, 0.07 spontaneous telomeric fusions/cell in two Artemis defective human cell lines (Fig 1B). The levels of spontaneous telomere fusions in Ku or DNA-PKcs defective cells were > 10 times higher [3]. This suggests that the effect of dysfunctional Artemis on telomeres in mammalian cells is much milder in comparison with Ku or DNA-PKcs defects Furthermore, we have demonstrated that (i) DNA damage occurring within the telomeric DNA is repaired less efficiently in "older" cells (PD 32+) irrespective whether they are Artemis defective or normal and (ii) Artemis defective "older" cells show higher proportion of DNA damage at telomeres than their normal counterparts (Fig. 5D). This means that the presence of a DSB within telomeric sequences (TIFs) causes a problem for repair mechanisms in "older" cells leading to differences in TIF frequencies 24 h after IR between "older" and "younger" cells. It has recently been shown that telomeric sequences pose a significant challenge to DNA replication machinery causing replication-dependent abnormalities very similar to aphidicolin-induced fragile sites [28]. The notion that telomeric sequences may behave as fragile sites originated from observations that interstitial telomeric sequences in Chinese hamster cells are prone to IR induced chromosome breakage [29]. Interestingly, Kruk et al. [30] found that UV induced damage at telomeres was repaired less efficiently than in transcribing DNA sequences but more efficiently that in other non-coding DNA sequences. More importantly Kruk et al. [30] found that DNA repair efficiency in cells from older donors was lower than in cells from younger donors. This is similar to our observation that TIFs persist at telomeres in "older" cells whereas "younger" cells repair IR induced damage at telomeres efficiently.

Finally, we have shown that DNA-PKcsi causes the greater effect on telomeres in Artemis defective cells than in control cells after approximately 45% reduction in DNA-PKcs expression (Fig 6). The greater effect of reduced DNA-PKcs expression on Artemis defective cells relative to control cells was confirmed by RNAi resulting in elevated frequencies of TIFs in Artemis defective cells 5 days after transfection. However, mechanisms behind the greater sensitivity of Artemis defective cells to telomere dysfunction relative to control cells remain unclear. It is worth pointing out that Artemis is involved in the repair of only a proportion of DSBs. For example, ~90% of IR induced DSBs are repaired relatively quickly and require Ku, DNA-PKcs and LIG-4/XRCC4 [20]. The remaining fraction of 10% DSBs are repaired by the combined action of ATM and Artemis [20] and they occur within heterochromatic regions of the genome [31]. Telomeres are typical heterochromatic parts of the genome [32,33]. It is therefore possible that Artemis and ATM are required specifically to repair of DSBs occurring within telomeric sequences. If this is true than we can argue that when Artemis is defective, as in the case of two cell lines used in this study, DSBs at telomeres cannot be repaired efficiently leading to cytological manifestation of telomere dysfunction (Fig. 1 and 2).

## Conclusion

We have shown that Artemis defect in human cell lines causes a mild telomere dysfunction phenotype detectable at the cytological level. However, mechanisms by which dysfunctional Artemis affects telomere maintenance remain unclear.

## Methods

### Cell culture and irradiation

The human primary fibroblast cell lines defective in Artemis, CJ179 and F01-240 were kindly provided by Prof. P.A. Jeggo, University of Sussex, Brighton UK. Details of mutations in each cell line have been described previously [20]. The normal human primary fibroblast, GM08399 was purchased from the Coriell Institute for Medical Research. All cell lines were cultured in D-MEM medium (Gibco/Invitrogen) supplemented with 10% fetal calf serum (Gibco/Invitrogen). The cultures were incubated at 37°C in the atmosphere of 10% CO_2_. Cells were irradiated, using a Cobalt^60 ^source (dose rate), at 80-90% confluency either in tissue culture flasks for chromosome analysis or on polyprep slides (Sigma) for TIF assay.

### Telo-FISH and Cytogenetic Analysis

Metaphase preparation was performed as described previously [34]. Briefly, cells were subjected to irradiation with gamma rays and incubated for 24 hours. The last 7 h of incubation 10 μl/ml of colcemid (10 μg/ml) (Sigma) was added to cell cultures followed by trypsinization, treatment with the hypotonic solution (5.6 g/l of KCl), fixation with methanol/acetic acid and slide preparation. Telo-FISH was performed as described previously [34]. Briefly, after appropriate washing slides were hybridized with the Cy-3 labelled telomeric PNA probe (CCCTAA)_3 _and left in a dark humidified chamber for 2 hours. After this slides were washed in 70% formamide and stained with the DAPI mounting medium (Vector Laboratories). Chromosomal analysis was performed using a Ziess-Axioplan2 microscope equipped with a CCD camera and MetaSystem image acquisition software (Imaging Associate).

### Telomere dysfunction induced foci (TIF) assay

The TIF assay described here is based on the detection of (i) DNA damage by an antibody against DNA damage marker γ-H2AX and (ii) telomeres by the synthetic PNA probe. Briefly, cells were incubated in tissue culture flask two days prior to experiment and after reaching a 80-90% confluency they were trypsinized and 1.0 ml of cell suspension (containing roughly 50,000 cells) was placed onto polyprep slides (Sigma) which were tranferred into petri dishes and left to grow for 24 h. Slides were then rinsed in PBS and fixed in 4% formaldehyde in PBS for 15 minutes. Cells were permeabilized in 0.2% (v/v) of Triton-X in distilled water at 4.0°C for 10 minutes and blocked with 0.5% (w/v) Bovine Serum Albumin (BSA) in PBS for 30 minutes. Anti-phospho-Histone H2A.X (Ser139) (Upstate) was diluted 1:500 with 0.5% BSA and 100 μl of resulting solution was added onto each slide and left to incubate for 1 hour in a damp container. After three washes in sterile tris-buffered saline tween-20 (TBS-T, pH7.4; 0.15 M NaCl, 0.268 mM KCl, 0.025 M tris-base, 500 μl/1l tween-20) for 3 minutes slides were incubated with 100 μl of secondary anti-goat antibody conjugated with Fluorescein isothiocyanate (FITC) and washed as above. After this slides were placed in 4% formaldehyde for 20 minutes for cross-fixing to preserve antibodies. The next step involved hybridization with telomeric PNA and this was performed as described above with the exception that TBS-T was used far washing instead of PBS.

### Western blotting

Western blotting has been carried out as described previously [35]. Briefly, cells grown in P100 petri dish were lyzed with 900 μl of lysis buffer (5× sample buffer; 10% (v/v) sodium dodecyl sulphate, 250 mM tris pH 8.0, 50% (v/v) glycerol, 0.01% (w/v) bromophenol blue). In addition, 50 μl of protease inhibitor (Roche) plus 50 μl of beta-mercaptoethanol was added and left for at least one minute. Cells were scraped off and collected in an eppendorf tube. Cells were then mechanically sheared ten times using a 1 ml syringe and a 23 g needle. Samples were spun at 13,000 RPM for 5 minutes at 4°C. Proteins were quantified using RC-DC protein assay (Bio-rad). Equal concentrations of 50 μg/50 μl were loaded onto a 4% precast gel (Bio-rad) until the high molecular weight protein marker (Invitrogen) was well separated. Following a wet blotting transfer to a polyvinylidine fluoride (PVDF) membrane and blocking with 5% (w/v) semi-skimmed milk (Marvel), the membrane was incubated with the mouse monoclonal primary antibody (DNA-PKcs, AB-4, Neomarker, Thermo Fisher Scientific) and β-acting (Abcam, Cambridge, UK) in a 1:500 and 1:1000 dilution respectively at 4°C overnight. This was followed by a secondary incubation period of 45 minutes with an HRP conjugated anti-mouse monoclonal rabbit antibody (Abcam, Cambridge, UK) diluted of 1:5000. The antibody detection was achived using chemiluminescence technique (ECL plus, GE healthcare) and protein levels were quantified using ImageQuant V5.1 (Molecular Dynamics, Piscataway, NJ).

### siRNA transfection

The two primary fibroblast cell lines (GM08399 and CJ179) were plated at a seeding density of 0.045 × 10^6 ^cells/1/2 ml roughly equating to 40% confluency in a 24-well plate. All siRNA transfections were done in duplicate using Dharmacon Accell SMARTpool siRNA reagent (Thermo Fisher Scientific, Lafayette. CO) targeting *PRKDC *gene (NM_006904) and a non-targeting (scrambled) siRNA as a negative control at a final concentration of 1 μM/μl and followed Dharmacon recommended protocol. Transfection was performed in a passive manner using a serum-free Accell transfection medium (Dharmacon). Cells were plated 24 h prior to transfection and incubated at 37°C in the incubator with the CO_2 _concentration adjusted to 10%. Total RNA were extracted at various time points as shown in Fig 6. The sequences of the human *PRKDC *gene siRNA were: UCUUGUGUUUAUUGGAUC, GGAAGAAGCUCAUUUGAUU, CGAUCAACACGGAAUUAUU and CUUUUAC AUAGCAUGGUUA as recommended by Dharmacon (Thermo Fisher Scientific, Lafayette. CO).

### Real-time quantitative PCR

A two step reverse-transcription PCR was performed using SYBR green 1 dye (Applied Biosystems) and expression of mRNA quantified in real-time with an ABI prism 7900HT sequence detection system (Applied Biosystems). The relative gene expression of DNA-PKcs were measured against the endogenous *GAPDH *gene and calculated using ΔCt. The sequence of DNA-PKcs and *GAPDH *primer were: forward 5'-CCGGACGGACCTACTACGACT-3' and reverse 5'-AGAACGACCTGGGCA TCCT-3', forward 5'-GAAGGTGAAGGTCGGAGT-3' and reverse 5'-GAAGATGG TGATGGGATTTC-3' respectively.

## Competing interests

The authors declare that they have no competing interests.

## Authors' contributions

HY carried out experiments. PS designed experiments with the help of HY. PS has written the manuscript. HY and PS have read and approved the final manuscript.

## References

[B1] Metcalfe JA Parkhill J Campbell L Stacey M Biggs P Byrd PJ Taylor AMR Accelerated telomere shortening in ataxia telangiectasia Nat Genet 1996 13 350 353 10.1038/ng0796-350 8673136

[B2] Hande MP Balajee AS Tchirkov A Wynshaw-Boris A Lansdorp PM Extra-chromosomal telomeric DNA in cells from Atm-/- mice and patients with ataxia-telangiectasia Hum Mol Genet 2001 10 519 528 10.1093/hmg/10.5.519 11181576

[B3] Bailey SM Meyne J Chen DJ Kurimasa A Li GC Lehnert BE Goodwin EH DNA double-strand break repair proteins are required to cap the ends of mammalian chromosomes Proceedings of the National Academy of Sciences of the United States of America 1999 96 14899 14904 10.1073/pnas.96.26.14899 10611310 PMC24745

[B4] Jaco I Munoz P Goytisolo F Wesoly J Bailey S Taccioli G Blasco MA Role of Mammalian Rad54 in Telomere Length Maintenance Mol Cell Biol 2003 23 5572 5580 10.1128/MCB.23.16.5572-5580.2003 12897131 PMC166323

[B5] Tarsounas M Muñoz P Claas A Smiraldo PG Pittman DL Blasco MA West SC Telomere Maintenance Requires the RAD51D Recombination/Repair Protein Cell 2004 117 337 347 10.1016/S0092-8674(04)00337-X 15109494

[B6] McPherson JP Hande MP Poonepalli A Lemmers B Zablocki E Migon E Shehabeldin A Porras A Karaskova J Vukovic B Squire J Hakem R A role for Brca1 in chromosome end maintenance Hum Mol Genet 2006 15 831 838 10.1093/hmg/ddl002 16446310

[B7] Slijepcevic P The role of DNA damage response proteins at telomeres--an "integrative" model DNA Repair 2006 5 1299 1306 10.1016/j.dnarep.2006.05.038 16798109

[B8] Sekiguchi JM Ferguson DO DNA Double-Strand Break Repair: A Relentless Hunt Uncovers New Prey Cell 2006 124 260 262 10.1016/j.cell.2006.01.010 16439201

[B9] d'Adda di Fagagna F Hande MP Tong W-M Roth D Lansdorp PM Wang Z-Q Jackson SP Effects of DNA nonhomologous end-joining factors on telomere length and chromosomal stability in mammalian cells Current Biology 2001 11 1192 1196 10.1016/S0960-9822(01)00328-1 11516951

[B10] Goodarzi AA Yu Y Riballo E Douglas P Walker SA Ye R Harer C Marchetti C Morrice N Jeggo PA Lees-Miller SP DNA-PK autophosphorylation facilitates Artemis endonuclease activity EMBO J 2006 25 3880 3889 10.1038/sj.emboj.7601255 16874298 PMC1553186

[B11] Poinsignon C de Chasseval Regina Soubeyrand Sebastien Moshous Despina Fischer Alain Robert J Haché G Villartay J-Pd Phosphorylation of Artemis following irradiation-induced DNA damage European Journal of Immunology 2004 34 3146 3155 10.1002/eji.200425455 15468306

[B12] Rooney S Alt FW Lombard D Whitlow S Eckersdorff M Fleming J Fugmann S Ferguson DO Schatz DG Sekiguchi J Defective DNA Repair and Increased Genomic Instability in Artemis-deficient Murine Cells J Exp Med 2003 197 553 565 10.1084/jem.20021891 12615897 PMC2193825

[B13] Cabuy E Newton C Joksic G Woodbine L Koller B Jeggo P Slijepcevic P Accelerated telomere shortening and telomere abnormalities in radiosensitive cell lines Radiation Research 2005 164 53 62 10.1667/RR3376 15966765

[B14] Lenain C Bauwens S Amiard S Brunori M Giraud-Panis M-Jp Gilson E The Apollo 52 Exonuclease Functions Together with TRF2 to Protect Telomeres from DNA Repair Cell 2006 16 1303 1310 10.1016/j.cub.2006.05.021 16730175

[B15] van Overbeek M de Lange T Apollo, an Artemis-Related Nuclease, Interacts with TRF2 and Protects Human Telomeres in S Phase Cell 2006 16 1295 1302 10.1016/j.cub.2006.05.022 16730176

[B16] Darroudi F Wiegant W Meijers M Friedl AA Burg M van der Fomina J van Dongen JJM van Gent DC Zdzienicka MZ Role of Artemis in DSB repair and guarding chromosomal stability following exposure to ionizing radiation at different stages of cell cycle Mutation Research/Fundamental and Molecular Mechanisms of Mutagenesis 2007 615 111 124 10.1016/j.mrfmmm.2006.11.029 17169382

[B17] Takai H Smogorzewska A de Lange T DNA Damage Foci at Dysfunctional Telomeres Cell 2003 13 1549 1556 10.1016/s0960-9822(03)00542-6 12956959

[B18] Harley CB Futcher AB CW G Telomeres shorten during ageing of human fibroblasts Nature 1990 345 458 460 10.1038/345458a0 2342578

[B19] Shay JW Wright WE Senescence and immortalization: role of telomeres and telomerase Carcinogenesis 2005 26 867 874 10.1093/carcin/bgh296 15471900

[B20] Riballo E Kuhne M Rief N Doherty A Smith GCM Recio Ma-J Reis C Dahm K Fricke A Krempler A Parker AR Jackson SP Gennery A Jeggo PA Löbrich M A Pathway of Double-Strand Break Rejoining Dependent upon ATM, Artemis, and Proteins Locating to gamma-H2AX Foci Molecular Cell 2004 16 715 724 10.1016/j.molcel.2004.10.029 15574327

[B21] Wang J Pluth JM Cooper PK Cowan MJ Chen DJ Yannone SM Artemis deficiency confers a DNA double-strand break repair defect and Artemis phosphorylation status is altered by DNA damage and cell cycle progression DNA Repair 2005 4 556 570 10.1016/j.dnarep.2005.02.001 15811628

[B22] Krempler A Deckbar D Jeggo PA Lobrich M An imperfect G2 M checkpoint contributes to chromosome instability following irradiation of S and G2 phase cells Cell Cycle 2007 6 1682 1687 17637566 10.4161/cc.6.14.4480

[B23] Bailey SM Brenneman MA Halbrook J Nickoloff JA Ullrich RL Goodwin EH The kinase activity of DNA-PK is required to protect mammalian telomeres DNA Repair 2004 3 225 233 10.1016/j.dnarep.2003.10.013 15177038

[B24] Ma Y Pannicke U Schwarz K Lieber MR Hairpin Opening and Overhang Processing by an Artemis/DNA-Dependent Protein Kinase Complex in Nonhomologous End Joining and V(D)J Recombination Cell 2002 108 781 794 10.1016/S0092-8674(02)00671-2 11955432

[B25] Zhang Y Zhou J Cao X Zhang Q Lim CUK Ullrich RL Bailey SM Liber HL Partial deficiency of DNA-PKcs increases ionizing radiation-induced mutagenesis and telomere instability in human cells Cancer letters 2007 250 63 73 10.1016/j.canlet.2006.09.021 17095151

[B26] Williams ES Klingler R Ponnaiya B Hardt T Schrock E Lees-Miller SP Meek K Ullrich RL Bailey SM Telomere Dysfunction and DNA-PKcs Deficiency: Characterization and Consequence Cancer Res 2009 69 2100 2107 10.1158/0008-5472.CAN-08-2854 19244120 PMC3595006

[B27] Musio A Veronica M Cristina S Francesca R Laura F Silvia G Gaetana L Luigi DN Domenico D Roberto C Vezzoni P Villa A Damaging-agent sensitivity of Artemis-deficient cell lines European Journal of Immunology 2005 35 1250 1256 10.1002/eji.200425555 15770702

[B28] Sfeir A Kosiyatrakul ST Hockemeyer D MacRae SL Karlseder J Schildkraut CL de Lange T Mammalian Telomeres Resemble Fragile Sites and Require TRF1 for Efficient Replication Cell 2009 138 90 103 10.1016/j.cell.2009.06.021 19596237 PMC2723738

[B29] Alvarez L Evans J Wilks R Lucas J Brown J Giaccia A Chromosomal radiosensitivity at intrachromosomal telomeric sites Genes Chromosomes Cancer 1993 8 8 14 10.1002/gcc.2870080103 7691162

[B30] Kruk P Rampino N Bohr V DNA damage and repair in telomeres: relation to aging Proceedings of the National Academy of Sciences 1995 92 258 262 10.1073/pnas.92.1.258 PMC42857 7816828

[B31] Goodarzi AA Noon AT Deckbar D Ziv Y Shiloh Y Löbrich M Jeggo PA ATM Signaling Facilitates Repair of DNA Double-Strand Breaks Associated with Heterochromatin Molecular Cell 2008 31 167 177 10.1016/j.molcel.2008.05.017 18657500

[B32] Garcia-Cao M O'Sullivan R Peters AHFM Jenuwein T Blasco MA Epigenetic regulation of telomere length in mammalian cells by the Suv39 h1 and Suv39 h2 histone methyltransferases Nat Genet 2004 36 94 99 10.1038/ng1278 14702045

[B33] Gonzalo S Blasco MA Role of Rb family in the epigenetic definition of chromatin Cell Cycle 2005 4 752 756 15908781 10.4161/cc.4.6.1720

[B34] Al-Wahiby S Slijepcevic P Chromosomal aberrations involving telomeres in BRCA1 deficient human and mouse cell lines Cytogenetics and Genome Research 2005 109 491 496 10.1159/000084208 15905643

[B35] Cabuy E Newton C Slijepcevic P BRCA1 knock-down causes telomere dysfunction in mammary epithelial cells Cytogenetics and Genome Research 2008 122 336 342 10.1159/000167820 19188703

